# Tuberculosis case finding based on symptom screening among immigrants, refugees and asylum seekers in Rome

**DOI:** 10.1186/1471-2458-13-872

**Published:** 2013-09-22

**Authors:** Monica Sañé Schepisi, Gina Gualano, Claudia Fellus, Nazario Bevilacqua, Marco Vecchi, Pierluca Piselli, Giuliana Battagin, Giulia Silvestrini, Andrea Attanasio, Alberto Vela, Giorgia Rocca, Alessandro Rinaldi, Pietro Benedetti, Salvatore Geraci, Francesco Nicola Lauria, Enrico Girardi

**Affiliations:** 1 Clinical Epidemiology Unit, Department of Epidemiology and Preclinical Research, National Institute for Infectious Diseases, IRCCS L. Spallanzani, Rome, Italy; 2 Respiratory Infectious Diseases Unit, Department of Clinical Research, National Institute for Infectious Diseases, IRCCS L. Spallanzani, Rome, Italy; 3 Local Health Unit AUSL RM D, Rome, Italy; 4 Caritas Health Service Network, Rome, Italy; 5 Local Health Unit AUSL RM H, STP Referral Centre, Nettuno (Rome), Italy; 6 "Salute per i migranti forzati" - SaMiFo – Health for forced migrants, Centro Astalli - Local Health Unit AUSL RM A, Rome, Italy

**Keywords:** Migrants, Tuberculosis, Active case finding, Adherence to screening, Access to care

## Abstract

**Background:**

In Italy the proportion of cases of tuberculosis in persons originating from high-prevalence countries has been increasing in the last decade. We designed a study to assess adherence to and yield of a tuberculosis screening programme based on symptom screening conducted at primary care centres for regular and irregular immigrants and refugees/asylum seekers.

**Methods:**

Presence of symptoms suggestive of active tuberculosis was investigated by verbal screening in migrants presenting for any medical condition to 3 free primary care centres in the province of Rome. Individuals reporting at least one symptom were referred to a tuberculosis clinic for diagnostic workup.

**Results:**

Among 2142 migrants enrolled, 254 (11.9%) reported at least one symptom suggestive of active tuberculosis and 176 were referred to the tuberculosis clinic. Of them, 80 (45.4%) did not present for diagnostic evaluation. Tuberculosis was diagnosed in 7 individuals representing 0.33% of those screened and 7.3% of those evaluated for tuberculosis.

**Conclusion:**

The overall yield of this intervention was in the range reported for other tuberculosis screening programmes for migrants, although we recorded an unsatisfactory adherence to diagnostic workup. Possible advantages of this intervention include low cost and reduced burden of medical procedures for the screened population. Further evaluation of this approach appears to be warranted.

## Background

Since the last two decades of the 20th century, migration from high tuberculosis incidence countries has contributed to slowing down [1] or even reverting [2] the decreasing trend in tuberculosis incidence in many European countries. Therefore interventions aimed at controlling tuberculosis in persons migrating from high incidence countries, including those based on active finding of individuals with active disease or latent infection, have been advocated as an important component of tuberculosis elimination strategies [3].

Many industrialised countries have put in place control strategies that have been mainly focused on screening new immigrants from countries with high rates of tuberculosis either before departure from the country of origin, or at entry into the host country [4-6]. More recently, interventions for migrants already resident in low incidence countries have been proposed. A study conducted in a district of London with high numbers of residents born outside the UK showed that promotion of verbal screening for tuberculosis in primary care practices improved identification of active tuberculosis [7]. Moreover there is evidence that eliciting the presence of the main tuberculosis symptoms (fever of more than one-week duration, cough of more than two weeks duration, night sweating, weight loss and haemoptysis) may represent a simple and sensitive method for screening of active tuberculosis, although this approach may have a low specificity [8,9].

The proportion of cases of tuberculosis among foreign born persons has considerably increased in Italy during the past decade passing from 22% in 1999 to 46% in 2008 [10], when this proportion was above 60% in major metropolitan areas such as in Rome area (Borgia P, Laziosanità, personal communication). This increase has paralleled the rise of the immigrant population in Italy from 1.5 million (2.7%) in 2002 to over 4.5 million (6.5%) in 2010 [11]. Moreover, half a million irregular immigrants were estimated to be present in Italy in 2010 [11] and, according to a national survey, irregular migrants account for slightly less than 50% of migrants hospitalised with tuberculosis [12]. However, no national policy for tuberculosis screening of migrants exists in Italy [4].

In this study we aimed at evaluating adherence to and yield of a screening programme for active tuberculosis based on verbal screening in primary care centres caring for regular and irregular immigrants and for asylum seekers in the province of Rome.

## Methods

### Study area and population

Three primary care centres for migrants in Rome province, Italy, two of them in the city of Rome and one in Nettuno, a town 60 km south of Rome, were used as recruitment sites. Study sites were: Caritas Health Service, a non-governmental out-patient clinic who provides primary and specialised medical care for regular and irregular immigrants; “SaMiFo” -Health for forced migrants-, a health service that mainly cares for asylum seekers and refugees, both in Rome; and an outpatient’s health clinic for irregular migrants in Nettuno, run by the Regional Health Service Unit.

All individuals presenting to these services between November 2009 and December 2010 were considered for inclusion in this study. Subjects were eligible if they were foreign-born and aged 18 or more. The study population included regular migrants, irregular migrants -defined as persons whose entry, stay or work in the country is illegal-, refugees and asylum seekers - defined as persons wishing to be admitted to the country as refugees and awaiting decision on their application- [13].

### Screening and diagnostic procedures

Eligible individuals underwent symptom screening which was performed through a standardised verbal questionnaire (Additional file 1) investigating on the presence of the following symptoms and signs: fever of more than one week duration, cough of more than two weeks duration, night sweating, weight loss, and haemoptysis. These symptoms were chosen because previous studies in settings with low incidence of HIV had shown that the presence of any of them provided 70% (95% CI 58–82) sensitivity in active tuberculosis screening programmes in adults [9]. In the same occasion, information on contact with tuberculosis patients, prior treatment for tuberculosis, BCG vaccination, and social and demographic information were collected.

Individuals reporting one of the previously listed symptoms/signs were referred to the tuberculosis clinic at the National Institute for Infectious Diseases L. Spallanzani (INMI) in Rome, for diagnostic workup. They were given instructions verbally and in writing on when and how to reach the tuberculosis clinic and they were informed that all examinations were free of charge; no support such as free transportation or monetary incentives was provided.

Patients reporting cough were instructed to carry an early morning sputum sample collected on the day of the visit. During the visit, a clinical examination and a chest radiograph were performed and a second sputum sample was collected. The individual returned after two-three days at the clinic, when an additional sputum sample was collected and further investigations were performed in selected cases if deemed necessary by the attending physician to confirm or rule out the diagnosis of tuberculosis.

For the purpose of the analysis, patients were considered as having completed diagnostic evaluation if they presented for the second visit at the tuberculosis clinic, and completed all the investigations considered necessary by the attending physician.

A case of tuberculosis was defined as a physician’s diagnosis of tuberculosis in a person who has bacteriological evidence of active disease and/or signs and symptoms compatible with tuberculosis and has completed diagnostic evaluation, and a physician’s decision to start treatment with a full course of antituberculosis chemotherapy [14].

The aims and methods of the study were explained to participating individuals in a printed leaflet written in eleven languages. The leaflets were handed to all participating individuals. Further information about the study was provided by cultural mediators, that also performed the interviews, in the patients’ own language for eight (Romanian, Bulgarian, Hindi, Amharic, Tigrinya, Farsi, Dari, Arabic) languages. Otherwise, Italian, French, or English were used as vehicular languages.

Cultural mediators were present at primary care centres when the questionnaire was administered.

Written consent was obtained from all subjects before enrolment. Ethical approval was provided by the Ethical Committee at INMI.

### Statistical analysis

Descriptive statistics are reported as proportions for categorical data or median (and interquartile range, IQR) for continuous variables. Chi-square test (or Fisher’s exact test when applicable) or Mann Whitney non-parametric test were used to compare groups respectively for categorical or continuous variables and to test their association. Data management and statistical analysis was performed using PASW-SPSS ver. 18 statistical package (SPSS Inc, USA).

For individuals reporting at least one tuberculosis symptom, we investigated the association with refusal or non-attendance to diagnostic evaluation of relevant characteristics using logistic regression analysis through odds-ratio (OR) and their 95% confidence intervals (95% CI). Gender, age and other variables found to be associated with refusal or non-attendance (p<0.1) were included in the multivariable logistic regression (MLR-OR) final model.

## Results

During the study period, 3350 individuals were seen at the three primary care centres. Among them, 2142 (63.9%) persons completed the interview and were enrolled in the study.

Characteristics of the individuals enrolled are summarised in Table 1. The median age of the participants (1551 males, 72.4%) was 32.3 years (IQR: 26.2-41.9). Overall, 92 nationalities were represented, among these the most frequently found were: Romania (293, 13.7%), India (220, 10.3%), Eritrea (190, 8.9%), Afghanistan (138, 6.4%), Nigeria and China (93 each, 4.3%). Many patients (44.8%) were from Africa. More than 60% came from countries with a tuberculosis incidence of 100 per 100.000 or higher. The median length of time spent in Italy was 2.1 years (IQR: 1.1-4.9): 47.2% of the subjects had spent less than 2 years in Italy; 33% 2– 5 years and 19.8% 6 years or more.

**Table 1:** Study population: demographic and social characteristics of 2142 screened individuals

		**All (n=2142)**	**Centres (a)**		
			**A (n=700)**	**B (n=951)**	**C (n=491)**
		**n (%)**	**n (%)**	**n (%)**	**n (%)**
Gender	Male	1551 (72.4)	584 (83.4)	616 (64.8)	351 (71.5)
Age (yrs)	Median (IQR)	32.3 (26.2-41.9)	28.9 (24.4-33.9)	38.0 (29.4-49.1)	32.2 (26.9-39.9)
Length of stay in Italy (yrs)	Median (IQR)	2.1 (1.1-4.9)	1.5 (1.0-2.4)	3.0 (1.3-6.8)	3.2 (1.2-5.8)
Education (yrs)	None/Not available	441 (20.6)	104 (14.9)	155 (16.3)	182 (37.1)
	1-8	725 (33.8)	290 (41.4)	341 (35.9)	94 (19.1)
	9+	976 (45.6)	306 (43.7)	455 (47.8)	215 (43.8)
	Median (IQR)	8 (6–13)	8 (5–12)	8 (8–13)	8 (8–12)
Geographic origin	Europe (b)	527 (24.6)	2 (0.3)	402 (42.3)	123 (25.1)
	Europe EU (pre-2004)+Western non EU	5 (0.2)	-	5 (0.5)	-
	New EU	404 (18.9)	-	308 (32.4)	96 (19.6)
	Other European countries	118 (5.5)	2 (0.3)	89 (9.4)	27 (5.5)
	Northern Africa	161 (7.5)	1 (0.1)	57 (6.0)	103 (21.0)
	Sub-Saharan Africa	799 (37.3)	548 (78.3)	211 (22.2)	40 (8.1)
	Latin America	61 (2.8)	1 (0.1)	54 (5.7)	6 (1.2)
	Asia	594 (27.7)	148 (21.1)	227 (23.9)	219 (44.6)
TB incidence rate in the country of origin (/10^5^) (c)	<25	151 (7.0)	12 (1.7)	108 (11.4)	31 (6.3)
	25-49	101 (4.7)	6 (0.9)	39 (4.1)	56 (11.4)
	50-99	424 (19.8)	158 (22.6)	190 (20.0)	76 (15.5)
	100-299	1176 (54.9)	292 (41.7)	558 (58.7)	326 (66.4)
	300+	290 (13.5)	232 (33.1)	56 (5.9)	2 (0.4)
Occupation	Yes	884 (41.3)	373 (53.3)	229 (24.1)	282 (57.4)
Migration pattern	Regular	568 (26.5)	6 (0.9)	462 (48.6)	100 (20.4)
	Irregular	710 (33.1)	3 (0.4)	319 (33.5)	388 (79.0)
	Asylum seeker/refugee	776 (36.2)	614 (87.7)	161 (16.9)	1 (0.2)
	Other/Not available	88 (4.1)	77 (11.0)	9 (0.9)	2 (0.4)
Place of residence	Apartment	1108 (51.7)	193 (27.6)	433 (45.5)	482 (98.2)
	Immigration centre	532 (24.8)	285 (40.7)	246 (25.9)	1 (0.2)
	Homeless/dormitories	466 (21.8)	221 (31.6)	240 (25.2)	5 (1.0)
	Not available	36 (1.7)	1 (0.1)	32 (3.4)	3 (0.6)
NHS registration (d)	Yes	804 (37.5)	699 (99.9)	105 (11.0)	0 (−)
Previous healthcare in Italy	Yes	577 (28.9)	259 (41.3)	228 (24.0)	60 (12.2)

(a) A: Public health service for asylum seekers/refugees; B: NGO health service for migrants; C: Public health service for migrants;

(b) According to EUROTB report classification: http://ecdc.europa.eu/en/publications/Publications/1103_TB_SUR_2009.pdf;

(c) According to WHO estimates: http://www.who.int/tb/publications/global_report/2010/en/index.html;

(d) Registration with the Italian National Health Service.

Seven hundred ninety-six (37.2%) subjects declared they never had a residence permit; 58.7% were not employed; 62.5% were not regularly registered with the Italian National Health Service mostly as not having right to register and 71.1% had had no contact with health services after their arrival in Italy, 64.7% had little or no knowledge of Italian language.

Among the individuals enrolled, 254 (11.9%) reported at least one symptom suggestive of active tuberculosis. Of them, 153 (60.2%) sought primary medical care due to symptoms other than protocol-defined tuberculosis symptoms. Among individuals with tuberculosis symptoms, 77 (30.3%) had prolonged cough alone and 135 (53.1%) cough associated with other symptoms.

**Figure 1:**
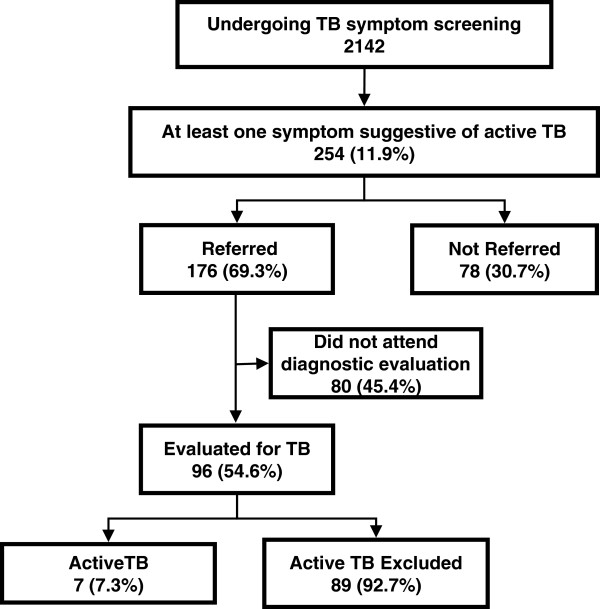
Flow diagram of the study.

Seventy-eight (30.7%) of individuals with protocol-defined tuberculosis symptoms were not referred for tuberculosis diagnostic evaluation, based on the judgement of the attending physician at the primary care centres (Figure 1).

Among the 1888 individuals not reporting protocol-defined tuberculosis symptoms, ten were referred for diagnostic evaluation: six of them were contacts of a tuberculosis case, one had a previous diagnosis of tuberculosis, one had a cervical lymph node enlargement, one had fever lasting less than seven days and one had chest pain.

Out of the 176 patients referred to the tuberculosis clinic, 80 (45.4%) did not present for diagnostic evaluation. We compared some factors (e.g., age, gender, birth country, length of stay in Italy) between those who attended the diagnostic evaluation and those who did not attend it. In multivariable analysis (Table 2), the probability of not attending tuberculosis clinic for diagnostic evaluation among symptomatic patients was higher in younger individuals and in irregular migrants or asylum seekers compared to regular migrants, and it also varied for patients seen in different primary care centres.

**Table 2:** Factors affecting refusal or non-attendance to diagnostic evaluation for tuberculosis among individuals with at least one symptom referred by primary care centres *

		**Total**	**Refusal or non-**	**OR (95% CI)**	**MLR-OR (95% CI)**
			**attendance**		
		**(n=176)**	**n (%)**		
All		176	80 (45.5)		
Gender	Male	143	62 (43.4)	1	1
	Female	33	18 (54.5)	1.6 (0.7–3.4)	1.9 (0.8–4.4)
Age (each 10–year increase)				0.8 (0.7–1.1)	0.7 (0.5–0.9)†
Length of stay in Italy (yrs)	<2	65	30 (46.2)	1	
	>2	106	49 (46.2)	1.0 (0.5–1.9)	
Centre (a)	A	39	8 (20.5)	0.2 (0.1–0.5)†	0.3 (0.1–1.1)‡
	B	108	59 (54.6)	1	1
	C	29	13 (44.8)	0.7 (0.3–1.5)	0.5 (0.2–1.2)
Tuberculosis incidence rate in the country of origin (/10^5^) (b)	<25	11	7 (63.6)	2.0 (0.5–7.9)	
	25–99	36	17 (47.2)	1	
	100+	129	56 (43.4)	0.9 (0.4–1.8)	
Occupation	No/Not available	131	61 (46.6)	1	
	Yes	45	19 (42.2)	0.8 (0.4–1.7)	
Legal status	Regular	120	48 (40.0)	1	1
	Irregular	56	32 (57.1)	2.0 (1.1–3.8)†	1.9 (0.9–4.1)
Place of residence	Apartment	83	38 (45.8)	1	
	Immigration centre	44	20 (45.5)	1.0 (0.5–2.1)	
	Homeless/dormitories	44	20 (45.5)	1.0 (0.5–2.1)	
	Other/Not available	5	2 (40.0)	0.8 (0.1–5.0)	
NHS registration (c)	No/Not available	126	67 (53.2)	1	1
	Yes	50	13 (26.0)	0.3 (0.2–0.6)†	0.8 (0.2–2.9)
Previous healthcare in Italy	No/Not available	125	64 (51.2)	1	1
	Yes	51	16 (31.4)	0.4 (0.2–0.9)†	0.7 (0.3–1.5)
Education (yrs)	None/Not available	38	19 (50.0)	1	
	1–8	62	28 (45.2)	0.8 (0.4–1.8)	
	9+	76	33 (43.4)	0.8 (0.4–1.7)	

* The sum could not add up to the total because of missing values;

OR: odds–ratio; MLR–OR: Multivariate–logistic regression odds ratio; CI: Confidence intervals †p<0.05; ‡p<0.10;

(a) A: Public health service for asylum seekers/refugees; B: NGO health service for migrants; C: Public health service for migrants;

(b) According to WHO estimates http://www.who.int/tb/publications/global_report/2010/en/index.html;

(c) Registration with the Italian National Health Service.

Overall, eight patients were diagnosed as active tuberculosis. Seven cases were diagnosed among individuals with protocol defined tuberculosis symptoms at screening, representing 0.33% (7/2142) of the individuals who underwent symptom screening, and 7.3% (7/96) of those who completed diagnostic evaluation. Among patients who completed diagnostic evaluation, tuberculosis was diagnosed in 1/55 (1.8%) reporting one or two symptoms and in 6/41 (14.6%) of those reporting 3 to 5 symptoms (p=0.04 by Fisher exact test).

Diagnosis of pulmonary tuberculosis was confirmed by sputum smear microscopy and culture in two individuals, and by DNA-PCR on the bronchoalveolar lavage fluid in one case, while it was based on clinical/radiological findings in the remaining four pulmonary cases.

Among the ten individuals referred for diagnostic evaluation without protocol-defined tuberculosis symptoms, five completed diagnostic evaluation and one extrapulmonary tuberculosis case was diagnosed by DNA-PCR assay on a lymph node aspirate.

All patients with a tuberculosis diagnosis started treatment, which was completed by five individuals, while the remaining three were lost to follow up before treatment completion.

## Discussion

The present study provides evidence that verbal symptom screening for tuberculosis of migrants in primary health care centres in a low incidence country, an intervention that requires limited resources, may have a yield that is in the range of most screening programmes for migrants.

Worldwide, a variety of different strategies for migrants tuberculosis screening are in use, and effectiveness of these interventions appears to be quite variable. In a systematic review of screening programmes in Europe, which included information on pre-entry, arrival and community post arrival screening, the coverage of the programmes ranged from less than 20% to almost 100%, and it was generally lower for voluntary screening and higher when directed to asylum seekers [13]. A variable coverage has also been observed in outreach programmes aimed at detecting active tuberculosis among disadvantaged populations in metropolitan settings. In London, the uptake of a radiographic screening conducted through a mobile unit in different settings varied from 30% to 90%, and a higher uptake was reported to be determined by the ability of local staff to engage pro-actively with clients, and providing small incentives and clear information [15].

In our study, among 254 individuals reporting at least one symptom suggestive of active tuberculosis, 30% were not referred for further diagnostic evaluation, mainly because the caring physician considered an alternative diagnosis more likely. Among those referred, almost half did not present to the tuberculosis clinic. The need to move to a different site for diagnostic evaluation may have contributed to the failure to complete evaluation. In a previous study conducted in Italy on active tuberculosis screening in undocumented migrants, the screening process was completed by 86% of them when chest X-ray was performed on site and in 70% of them when they were referred to another service for radiographic evaluation [16].

In the population studied, the probability of not attending diagnostic evaluation was lower for younger patients and tended to be higher for males and for irregular migrants. It was different in the three sites involved, although these differences were not statistically significant at the conventional 0.05 level. It has to be noted that the service with the lowest recorded rate of adherence to the screening procedures is a busy primary care clinic that provides care mainly to disadvantaged people including homeless, drug-abuser, alcoholic and extremely indigent people in addition to regular and irregular migrants. To try to increase completion of screening, the tuberculosis clinic staff tried to contact directly by phone individuals which did not show up for diagnostic evaluation and provided information of non-attendance to the participating centres, that could in turn reinforce the invitation to adhere to their clinic appointment. These data suggest that additional strategies are needed to increase the effectiveness of this intervention. These may include being interviewed by a health care worker in the same native language [17], active involvement of peers [18,19], brief educational programmes [20-22] and the provision of small monetary incentives that have proven effective in favouring the acceptance of tuberculosis screening in other population groups [23,24].

In spite of the sub optimal adherence to diagnostic procedures, the overall yield of our programme (0.33%) was in the range reported for other tuberculosis screening programmes for migrants. In a meta-analysis of screening programmes at port of entry, the overall yield of pulmonary tuberculosis was 3.5 cases per thousand screened, and this figure tended to be higher for asylum seekers and for individuals originating from Asia or Africa [25]. Similarly, in the systematic review [13] of 14 national screening programmes in the European Union, a median yield of 1.8 per thousand has been recorded. Neither study provided evidence of superior effectiveness of any of the approaches to screening used.

The main difference between the programme described in the present paper and the vast majority of the tuberculosis screenings directed at new immigrants is that we did not include any laboratory or imaging tool in the first part of the screening, and these diagnostic interventions were only applied to those reporting at least one symptom suggestive of active tuberculosis. This approach is attractive because it may significantly reduce the use of resources and it may be applied when radiographic facilities are not available at the sites where target population is first screened. On the other hand, we cannot rule out that some prevalent tuberculosis cases were not identified by verbal screening. A series of studies, conducted in resource-constrained settings with high HIV prevalence, which evaluated the sensitivity of symptom based tuberculosis screening, has shown that a rule based on the presence of at least one symptom (current cough, fever, night sweats, or weight loss) had an overall sensitivity of 78.9%, and chest radiograph increased this sensitivity by 11.7% [8]. A symptom-based approach has also been used to screen for tuberculosis among asylum seekers in Switzerland since 2006. This approach was estimated to have a 55% sensitivity compared to a 100% sensitivity of the radiographic screening carried out until 2005 [26]. Thus, available evidence suggests that sensitivity of a symptom-based screening may be significantly lower than sensitivity of “traditional” radiographic screening.

The study design did not allow measuring to what extent verbal symptom screening may have increased the detection rate of active tuberculosis and/or reduced diagnostic delay compared to traditional passive case finding. However, a study conducted in London provides evidence that education of primary care providers to perform verbal screening for tuberculosis not only increased identification of people with latent tuberculosis but also of those with active tuberculosis [7]. Moreover, there is evidence that active case finding based on repeated symptom screening may be effective in reducing prevalence of active tuberculosis and tuberculosis transmission in high burden settings [27]. It can be also hypothesised that repeated screening may increase awareness of tuberculosis symptoms and the likelihood of seeking care for tuberculosis among those developing tuberculosis related symptoms.

The diagnosis of pulmonary tuberculosis has been confirmed by microbiological examinations only in three of the seven patients observed in the programme. We can speculate that this low rate of microbiological confirmation of the diagnosis could be attributed at least in part to the fact that active screening may allow the detection of tuberculosis at an earlier stage, with a lower mycobacterial burden, compared to passive case finding. This is in line with previous studies suggesting that active screening is associated with a reduction in the severity or infectivity of identified cases, with a lower proportion of cases who were symptomatic or smear or culture-positive [28-30].

On the other hand, we cannot rule out a diagnostic bias due to the inclusion in a tuberculosis screening programme.

Another limitation of the study is that we do not have information on the prevalence of HIV infection in the population studied, which may influence the clinical presentation of tuberculosis [31]. However, in the context of active case finding there is no evidence of a different sensitivity of symptom-based screening in HIV- infected and non-infected persons [32,33].

## Conclusion

Interventions to control tuberculosis in migrants are badly needed in Europe and, in this context, initiatives to favour early access to care should be put in place [34,35].

Our study suggests that a non-negligible number of tuberculosis in migrants cases may be identified trough verbal symptom screening in primary care centres, and then linked to care: this approach may thus complement the widely used radiographic screening of migrants at entry into the country. Possible advantages of this intervention include low cost, reduced burden of medical procedures for the screened population, possibility to be repeated over time after migration and potential to reach different groups of migrants.

Further studies should assess tools aimed at increasing adherence to this intervention and its feasibility in different settings where medical care for migrants is provided.

## Competing interests

The authors declare that they have no competing interests.

## Authors’ contributions

EG conceived of the study and its design, and coordinated it, with the collaboration of MSS and CF. PP was in charge of the database and performed the statistical analysis. All of them drafted the manuscript, with specific contributions. GB, GG, NB and MV, under the supervision of FNL performed the diagnostic workup. AA and GS, under the supervision of SG, enrolled study subjects and administered the symptom questionnaire -at Caritas Health Service, as GR and AR, did, under the supervision of PB at "SaMiFo" - Health for forced migrants, AUSL RM A, and as AV did at “STP Referral Centre Nettuno”. All Authors reviewed earlier versions of the manuscript, read and approved the final manuscript.

## Pre-publication history

The pre-publication history for this paper can be accessed here:

http://www.biomedcentral.com/1471-2458/13/872/prepub

## Supplementary Material

Additional file 1 Tuberculosis symptom screening questionnaire. Click here for file
