# Supplementary material for: Tuberculosis case finding based on symptom screening among immigrants, refugees and asylum seekers in Rome
Source: BMC Public Health. 2013 Sep 22;13:872. doi: 10.1186/1471-2458-13-872 (PMC3852535; doi:10.1186/1471-2458-13-872)
Supplement: Additional file 1 — Tuberculosis symptom screening questionnaire. [file 1471-2458-13-872-S1.pdf]

ID code|__|__|__|__|__|__|__|

(Formed by:

- *recruitment site: letter A (RMA), H (RMH), C (Caritas)*
- *enrollment number(4 digits), independent for each recuitment site*
- *access number (2 digits) (confirmed by identity check)*

Year of birth |__|__|__|__|

Sex |_1_| M |_2_| F

Contry of birth ______________________

Nationality ______________________

Education *(years of schooling, not applicable if younger than 6 years of age)* |__|__|

Date first arrival in Italy (mm/yyyy) |__|__/|__|__|__|__|

Residence permit *(main reason for issuing)*

|_1_| Regular employment |_2_| Family reasons |_3_| Study |_4_| Humanitarian grounds |_5_| International protection |_6_| Refugee |_7_| Medical reasons |_8_| Religious worker|_9_| Tourist visa (valid) |_10_| Other|_11_| Awaiting renewal |_12_| Expired |_13_|never had a residence permit

Main present occupation (*not applicable if younger than 15 years of age*)

|_1_| Street seller |_2_| Self-employed (sales person, craftsman) |_3_| Employee |_4_| Caretaker, Baby-sitter |_5_| Housekeeper |_6_| Health care worker |_7_| Student|_8_| Restaurant staff |_9_| Farmer |_10_| Construction worker |_11_| Industry worker |_12_| Housewife |_13_| Other |_14_| Unemployed

Knowledge of italian language

(evaluated by the interviewer)

none/little/fair

Presently lives *(there can be more than one answer)*

|_1_| Privately owned apartment |_2_| Rented apartment |_3_| Apartment owned by company/boss |_4_| Place of work |_5_| Daycare center, homeless shelter |_6_| Abandoned building (squatter) |_7_| hut/caravan/tent |_8_| Homeless |_9_| Other

B) CLINICAL PATHWAYS

Registration with the Italian National Health Service **YES|_1_|** **NO|_2_|**

If NOT, why?

|_1_| In progress **|_2_|** Financial or burocratic difficulties **|_3_|**Not having the right to register **|_4_|** Cultural and language difficulties

|_5_| Lack of information **|_6_|** Other

STP Code |__|__|__|__|__|__|__|__|__|__|__|__|__|__|__|__|

Previous contact with health services after arrival in Italy? YES|_1_| NO|_2_|

If YES, indicate the date of the first contact (mm/ yyyy) **|__|__/|__|__|__|__|**

C) HEALTH INFORMATION

Medical record number|__|__|__|__|__|__|__|__|__|__|__|__|__|__|__|__|

Interview/physical examination date (dd/mm/yyyy)

|__|__/|__|__/|__|__|__|__|

Diagnosis or clinical suspicion

(description of the diagnosis or if lacking, of the main reason for seeking care)

____

____

TB SYMPTOMS SCREENING

Have you ever been vaccinated with BCG (TB vaccine)?
|__| YES **|__| NO** **|__|** Does not remember

If YES indicate the date of vaccination: (dd/mm/yyyy) **|__|__/|__|__/|__|__|__|__|**

Have you ever had tuberculosis (TB)?
|__| YES **|__| NO** **|__|** Does not remember

If YES indicate the date of diagnosis: (dd/mm/yyyy) **|__|__/|__|__/|__|__|__|__|**

If YES it was |__| pulmonary TB **|__|** extrapulmonay TB

Have you ever had contact with someone with active tuberculosis?
|__| YES **|__| NO** **|__|** Does not remember

If YES indicate the date of the last contact:

(dd/mm/yyyy**)|__|__/|__|__/|__|__|__|__|**

If YES it was a |__| household contact **|__|**close contact

Have you been coughing for more than 2 weeks?
|__| YES **If YES since when?:** (dd/mm/yyyy) **|__|__/|__|__/|__|__|__|__|**

|__| NO

Have you had a fever for more than 7 days? (reported every day for the past 7 days)
|__| YES   If YES since when?: (dd/mm/yyyy) **|__|__/|__|__/|__|__|__|__|**

|__| NO

Have you recently experienced night sweats?
|__| YES   If YES since when?: (dd/mm/yyyy) **|__|__/|__|__/|__|__|__|__|**

|__| NO

Have you been losing weight (> 10%) recently?
|__| YES   If YES since when?: (dd/mm/yyyy) **|__|__/|__|__/|__|__|__|__|**

|__| NO

Have you recently been coughing up blood?
|__| YES   If YES since when?: (dd/mm/yyyy) **|__|__/|__|__/|__|__|__|__|**

|__| NO

If reporting at least one of the previously listed symptoms/signs, individuals should be referred to the tuberculosis clinic at the National Institute for Infectious Diseases L. Spallanzani (INMI) in Rome, for diagnostic workup.

In case of a clinical suspicion of TB or if immunodepressed subjects, referral to the tuberculosis clinic at INMI is recommended.

PATIENT REFERRED TO DIAGNOSTIC WORKUP AT INMI:
|__| YES |__| NO
